# Value of troponin T as a screening test of cardiac structure and function in chronic kidney disease

**DOI:** 10.21542/gcsp.2021.26

**Published:** 2021-12-31

**Authors:** Fatma M. Nasr, Amna Metwaly, Ashraf Abdel Khalik, Manar Raafat, Malak Nabil, Laila Kamel, Noha Elsheikh

**Affiliations:** 1 Intensive Care Department, Theodor Bilharz Research Institute (TBRI), Jizah, Egypt; 2 Nephrology Department, Theodor Bilharz Research Institute, Jizah, Egypt; 3 Clinical Chemistry Department, Theodor Bilharz Research Institute, Jizah, Egypt

## Abstract

Background: Cardiovascular disease starts early in the course of chronic kidney disease (CKD) and is the leading cause of death in patients with end-stage renal disease. Since high-sensitivity cardiac troponin T (hs-cTnT) can detect much lower levels of myocardial injury than conventional assays, it may be useful for studying the earliest stages of heart disease in patients with CKD.

Objective: To evaluate the association of circulating hs-cTnT with LV structural and functional abnormalities detected by echocardiography among dialysis dependent and non-dialysis dependent CKD patients.

Methods: This study was conducted on 107 subjects divided into three groups.

Group I consisted of CKD patients on conservative treatment (*n* = 42), Group II: hemodialysis patients (*n* = 42), Group III: control group: age and sex matched healthy volunteers (*n* = 23). All subjects were subjected to clinical examination, biochemical evaluation including estimation of hs-cTnT and Echo-Doppler study of cardiac structure and function.

Results: There was a significant increase in LAV (*p* < 0.01), LVM (*p* < 0.01) in both patient groups compared to the control group. Mitral annular plane systolic excursion (MAPSE) was significantly decreased in both patient groups compared to the control group (*p* < 0.01, *p* < 0.05) and in group I compared to group II (*p* < 0.05) with a significant decrease in S velocity in group I compared to groups II and III (*p* < 0.01). There was a significant decrease in Vp (*p* < 0.01) with a significant increase in AEF (*p* < 0.01) in both patients’ groups compared to the control group and AEF was significantly increased in group II compared to group I (*p* < 0.01). Ea velocity and Ea/Aa decreased significantly (*p* < 0.01) with significant increase in Aa velocity (*p* < 0.05, *p* < 0.01), E/Ea (*p* < 0.01) and E/Vp (*p* < 0.05) in both patient groups compared to the control group.

There was a significant increase in hs-cTnT levels in both patient groups compared to the control group (*P* < 0.01). We found a positive correlation between hs-cTnT levels and LAV (*r* = 0.291, *p* < 0.03), IVST (*r* = 0.374, *p* < 0.004), PWT (*r* = 0.309, *p* < 0.02), LVM (*r* = 0.282, *p* < 0.03), A wave velocity (*r* = 0.271, *p* < 0.04), E/Ea (*r* = 0.506, *p* < 0.0001), PCWP (*r* = .507, *p* < 0.0001) and a negative correlation between hs-cTnT and MAPSE (*r* =  − 0.300, *p* < 0.02), S wave velocity (*r* =  − 0.259, *p* < 0.05), Ea (*r* =  − 626, *p* < 0.0001), Ea/Aa (*r* =  − 0.543, *p* < 0.0001).

Troponin at the cut-off value of >5 ng/L, revealed 100% sensitivity and 95% specificity with areas under curve (AUC) of 0.998 and accuracy of 95.65% (*P* < 0.01) for discrimination of Group I vs control group and 76.2% sensitivity and 95.7% specificity with AUC 0.796 and accuracy 71.84% (*P* < 0.01) for discrimination of group II vs control group.

Conclusion: Structural and functional cardiac abnormalities are common in CKD patients. Serum hs-cTnT levels increased in CKD patients and was associated with LVH, LAV and some of the echocardiographic parameters of LV systolic and diastolic dysfunction.

Our research suggests that hs-cTnT levels may be important for early screening of cardiac structure and function in CKD patients to provide evidence for early intervention.

## Introduction

The major cause of death in patients with end-stage renal disease (ESRD) is cardiovascular disease (CVD), which begins early in the course of chronic kidney disease (CKD)^1^. Left ventricular hypertrophy (LVH), left ventricular (LV) dilatation, and cardiac systolic or diastolic dysfunction are present in most CKD patients starting dialysis, predisposing them to an increased risk of ischemic heart disease (IHD), heart failure, or cardiac death^2^.

Echocardiographic studies may play a critical role in evaluating cardiac structure and function. Two-dimensional (2D) and M-mode echocardiographic images are capable of assessing LV geometry, quantifying LV mass and systolic function^3^. Doppler imaging can provide indirect information about LV relaxation and diastolic function. The assessment of LV function has been improved by the use of tissue Doppler imaging (TDI)^4^.

Many traditional cardiac risk factors, as well as non-traditional risk factors specifically associated with renal disease (LVH, anemia, hyperparathyroidism, altered calcium–phosphate metabolism and others), can be modified by aggressive therapy. In addition, detecting cardiac disease at an early stage would facilitate aggressive treatment of patients at increased risk^5^. Therefore, it is important to investigate serum biomarkers for early assessment of cardiac structure and function in CKD patients, to provide evidence for early intervention.

Cardiac troponin T (cTnT) and troponin I (cTnI) are integral parts of cardiac muscle infrastructure and play a major role in excitation-contraction coupling^6^.

Cardiac myocyte membrane damage leads to the release of cardiac troponins into circulation, which can be detected by specific immunoassays developed for these proteins (TnT and cTnI)^7^.

New high-sensitivity cardiac troponin (hscTnT) assays have been developed with detection limits 10–100 times lower than those detected by traditional methods^8^. Previous studies reported that the level of serum hscTnT was higher in CKD patients than in non-CKD patients^9^. Some studies have suggested that hscTnT can be used to evaluate cardiac structure and function abnormalities in CKD patients^9–13^. However, these studies are sparse and have led to different conclusions.

Therefore, we carried out the following research to evaluate the association of circulating hs-cTnT with LV structural and functional abnormalities, as detected by echocardiography, among dialysis-dependent and non-dialysis-dependent CKD patients.

## Patients and methods

### Patients

This study was conducted on 107 subjects (84 CKD patients recruited from the Nephrology Department at Theodor Bilharz research institute, in addition to 23 age- and sex-matched healthy volunteers). The subjects were divided into three groups:

• Group I: Patients with CKD on conservative treatment (*n* = 42).
• Group II: Patients with CKD on regular hemodialysis (HD) (*n* = 42), 3x per week in 4-hour sessions for more than 6 months.
• Group III: control group (*n* = 23).

Demographic and clinical data were recorded including age, sex, duration of dialysis, heart rate, and blood pressure.

Exclusion criteria were history of rheumatic heart disease, congenital heart disease, myocardial infarction, cardiomyopathy, atrial fibrillation, uncontrolled hypertension, clinical signs of heart failure, and severely anemic patients (Hb<10g/dl).

Written informed consent was obtained from all patients. The study protocol was in accordance with the declaration of Helsinki 1975 as modified in 2012, and approved by the institutional review board of TBRI before enrolling participants.

### Laboratory investigations

Routine laboratory investigations including kidney function tests, serum electrolyte and lipid profiles were assayed with a Beckman Coulter AU 480 autoanalyzer (Beckman Coulter, Brea, California, USA). A complete blood picture was assayed with Swelab Alpha Plus (Boule Diagnostics AB Domnarvsgatan, Sweden).

High-sensitivity cardiac troponin T was determined using the cTnT 4th-generation electrochemiluminescent immunoassay “ECLIA” on Cobas e411 immunoassay autoanalyzer according to the instructions of the manufacturer (Roche Diagnostics GmbH, Mannheim, Germany). The analytical measurement range was 3 to 10.000 pg/mL.

In the hemodialysis group (II), blood samples were taken before the dialysis session.

### Echo-Doppler study

Echo-Doppler was performed on all subjects according to ASE/EACVI recommendations^14,15^ by two members of the study team using a high resolution (x11-15305) Sonata Plus ultrasound scanner.

• Measurements of the dimensions of the left ventricle and its walls were performed in the parasternal long-axis view at or immediately below the level of the mitral valve leaflet tips. LV mass was calculated using the Devereux formula:

LV mass(g) = 0.8  × 1.04  × [LVIDd + PWTd + IVSd]^3^ − [LVID]^3^ + 0.6^16^.

Where LVIDd is the left ventricular end-diastolic dimension, PWTd is the end diastolic posterior wall thickness, IVSd is the end diastolic interventricular septal thickness.

• LV volumes were measured in the apical four- and two-chamber views using the biplane method of disks (modified Simpson’s rule) summation and LV ejection fraction was estimated.
• Mitral annular diameter was measured from apical four chamber view and mitral orifice area was calculated assuming that it is circular. Mitral orifice area= *π* (Mitral annular diameter/2)^2^.
• Left atrial dimensions were measured. Measurement of left atrial volume was done from apical 4-chamber and apical 2-chamber views at ventricular end-systole using biplane method of disks (modified Simpson’s rule).
• Mitral annular plane systolic excursion (MAPSE) was measured in an apical four chamber view from the lowest point to the highest point during systole using M-mode echocardiography. MAPSE was measured from the septal and lateral mitral annulus and measurements were averaged.
• The Left Ventricular Outflow Tract was used to estimate stroke volume and cardiac output (COP) from the following equation^17^: “Stroke Volume = LVOT VTI  × Cross Sectional Area of LVOT”. (LVOT: Left Ventricular Outflow Tract, VTI: Velocity Time Integral.)
  – COP = stroke volume ×  heart rate.
  – LVOT VTI was calculated by placing the pulsed Doppler sample volume in the outflow tract below the aortic valve and recording the velocity (cm/s) in the apical four-chamber. LVOT diameter measurement was made just below the aortic valve plane in mid-systole in the parasternal long-axis view^18^. “Cross Sectional Area of LVOT = *π* (LVOT diameter/2)^2^.”
  – Pulsed Doppler was used to record transmitral flow in the apical four chamber view. The sample volume was placed at the tip of the mitral valve. From the mitral valve inflow velocity curve, we measured: peak E wave velocity (cm/s), peak A wave velocity (cm/s), E/A ratio and deceleration time (DT, ms). Isovolumic relaxation time (IVRT, ms) which represents the time interval from aortic valve closure to mitral valve opening, was measured using a continuous wave Doppler signal, which intersects both the left ventricular outflow and the mitral valve motion.
  – Tissue Doppler velocities were measured at the lateral and medial sites of the mitral annulus and measurements were averaged. We measured peak early diastolic mitral annular velocities (Ea, cm/s) and peak late diastolic mitral annular velocities (Aa, cm/s) and Ea/Aa was calculated.
  – Flow propagation velocity (Vp) (cm/s) was measured from color M mode in the apical four chamber view by measuring the slope of the early diastolic color m-mode wave.
  – LV filling pressure was estimated from E:Ea and E:Vp ratios^19,20^.
  – Pulmonary capillary wedge pressure (PCWP) was calculated according to the formula: “PCWP = 1.55 + 1.47(E/Ea)”^21^.
  – We measured the mitral peak systolic annular velocity (Sa) at the two annular sites and measurements were averaged.
  – LA ejection force was calculated as 0.5 x 1.06 x mitral annular area x (peak A velocity)^2^ in kdynes^22^.
  – Identification of diastolic dysfunction was performed according to ASE/EACVI recommendations^15^. The ASE/EACVI recommended four variables for identification of left ventricular diastolic dysfunction (LVDD) and their abnormal cut-off values in subjects with normal LVEF: annular e’ velocity (septal e’ < 7 cm/s, lateral e’ < 10 cm/s), average E/e’ > 14, LA volume index > 34 mL/m2, and peak TR velocity > 2.8 m/s. Left ventricular diastolic function was normal when less than half of the available variables met the cut-off values for identifying abnormal function. LVDD was positive when more than half of the available variables met these cut-off values, but was indeterminate when only half met these values. Patients were graded in three groups (normal diastolic function, indeterminate diastolic function, and positive diastolic dysfunction).
  – Echocardiographic studies of the patients on dialysis were performed before dialysis.

## Statistical Analysis

Data analysis was performed using Microsoft Excel 2016 and Statistical Package for Social Science (SPSS) Statistics for Windows, version 26 (IBM Corp., Armonk, N.Y., USA). Data was expressed as the mean ± SD. *P* ≤ 0.05 was considered statistically significant, and *p* ≤ 0.01 was considered highly significant.

Pearson correlation coefficient was calculated to get the association between hs-cTnT and different variables.

The diagnostic performance of Troponin, LA volume, IVST, LVPWT, LV mass, MAPSE, A, Ea avg, Ea/Aa avg, E/Ea avg, PCWP, and S avg was assessed by receiver operating characteristic (ROC) curves. The area under the ROC (AUC) was calculated as an accuracy index for prognostic performance of selected tests. The cutoff for the diagnosis of a group of the study was taken from the point of maximum combined sensitivity and specificity.

**Table 1:** Demographic data of the studied groups.

Variables	Group I (*N* = 42) Mean ± SD	Group II (*N* = 42) Mean ± SD	Group III (*N* = 23) Mean ± SD	P1 1&3	P2 2&3	P3 1&2
Age (year)	54.90 ± 11.13	50 ± 13.73	51.55 ± 8.71	NS	NS	NS
Gender N (%) Male Female	19 (45.24%) 23 (54.76%)	22 (52.38%) 20 (47.62%)	10 (43.48%) 13 (56.52%)	NS	NS	NS
Duration of dialysis (years)		7.3 ± 4.39				
Pulse (beats/min)	89.19 ± 9.22	86.09 ± 8.62	82.69 ± 6.02	<0.01	NS	NS
SBP (mmHg) DBP (mmHg)	144.8 ± 18.7 83.35 ± 12.4	140.78 ± 17.2 81.19 ± 12.53	125.6 ± 8.91 74.4 ± 8.6	<0.01 <0.01	<0.01 <0.05	NS NS

**Notes.**

N number SBP systolic blood pressure DBP diastolic blood pressure P1 value between groups 1&3 P2 value between groups 2&3 P3 value between groups 1&2 NS not significant

**Table 2:** Laboratory data of the studied groups.

Variables	**Group I** (*N* = 42) Mean ± SD	**Group II** (*N* = 42) Mean ± SD	**Group III** (*N* = 23) Mean ± SD	*P* value		
				1&3	2&3	1&2
Urea	144.60 ± 55.98	167.00 ± 66.44	16.35 ± 3.20	<0.01	<0.01	NS
Creatinine	4.36 ± 2.14	8.28 ± 1.29	0.77 ± 0.17	<0.01	<0.01	<0.01
GFR	17.11 ± 9.96	6.38 ± 1.47	98.43 ± 18.94	<0.01	<0.01	<0.01
Cholesterol	137.40 ± 56.51	142.52 ± 22.13	116.61 ± 20.21	NS	<0.01	NS
Triglycerides	108.34 ± 55.64	129.05 ± 21.40	87.04 ± 13.98	NS	<0.01	<0.05
Calcium	7.76 ± 1.19	8.19 ± 0.88	9.42 ± 0.62	<0.01	<0.01	NS
Phosphorous	5.33 ± 1.32	5.27 ± 1.4	3.63 ± 0.74	<0.01	<0.01	NS

**Notes.**

GFR Glomerular filtration rate

**Table 3:** Echocardiographic data of the studied groups.

Variables	**Group I** (*N* = 42) Mean ± SD	**Group II** (*N* = 42) Mean ± SD	**Group III** (*N* = 23) Mean ± SD	*P* value		
				1&3	2&3	1&2
LAV (mL)	48.67 ± 12.25	50.12 ± 11.30	39.30 ± 6.15	<0.01	<0.01	NS
Mitral annular Diameter (cm)	2.94 ± 0.39	3.00 ± 0.51	2.84 ± 0.31	NS	NS	NS
AO (mm)	30.14 ± 4.70	30.33 ± 4.69	27.43 ± 3.29	<0.05	<0.05	NS
IVST (cm)	1.17 ± 0.17	1.13 ± 0.14	1.00 ± 0.13	<0.01	0.01	NS
PWT (cm)	1.15 ± 0.15	1.16 ± 0.16	1.01 ± 0.11	<0.01	<0.01	NS
LVIDd (cm)	5.11 ± 0.52	5.25 ± 0.69	4.86 ± 0.43	NS	<0.05	NS
LVIDs (cm)	3.24 ± 0.57	3.23 ± 0.67	2.91 ± 0.47	<0.05	<0.05	NS
LVM (gm)	232.76 ± 45.05	243.66 ± 73.67	178.17 ± 47.16	<0.01	<0.01	NS
FS %	37.86 ± 5.43	38.33 ± 7.69	40.10 ± 7.59	NS	NS	NS
EF %	67.19 ± 6.79	67.38 ± 9.80	70.43 ± 8.36	NS	NS	NS
LVOTD (cm)	2.11 ± 0.24	2.23 ± 0.35	1.97 ± 0.12	<0.05	<0.01	NS
LVOT VTI (cm)	20.52 ± 3.43	23.36 ± 4.32	20.22 ± 1.59	NS	<0.01	<0.01
SV (mL)	72.09 ± 17.84	93.43 ± 33.91	62.54 ± 12.32	<0.05	<0.01	<0.01
COP (L/min)	6.40 ± 1.58	8.02 ± 3.00	5.19 ± 1.17	<0.01	<0.01	<0.01
MAPSE (mm)	13.62 ± 1.96	14.95 ± 2.77	16.61 ± 2.25	<0.01	<0.05	<0.05
E velocity (m/s)	68.53 ± 22.50	71.24 ± 24.43	68.61 ± 6.63	NS	NS	NS
A velocity (m/s)	75.43 ± 17.92	87.57 ± 24.44	53.27 ± 7.35	<0.01	<0.01	<0.01
E/A	0.96 ± 0.41	0.84 ± 0.28	1.32 ± 0.25	<0.01	<0.01	NS
DT (ms)	142.95 ± 34.40	166.71 ± 63.92	154.52 ± 13.27	NS	NS	<0.05
IVRT (ms)	69.76 ± 10.57	71.43 ± 11.33	69.39 ± 12.77	NS	NS	NS
LV Vp (cm/s)	43.00 ± 12.11	45.00 ± 7.12	51.52 ± 6.28	<0.01	<0.01	NS
AEF (Kdynes)	21.43 ± 10.11	31.98 ± 19.74	9.65 ± 2.66	<0.01	<0.01	<0.01
Ea avg (cm/s)	8.52 ± 1.70	8.84 ± 2.16	13.09 ± 1.26	<0.01	<0.01	NS
Aa avg (cm/s)	10.80 ± 2.02	12.13 ± 2.82	9.54 ± 1.84	<0.05	<0.01	<0.05
Ea/Aa avg	0.84 ± 0.20	0.78 ± 0.18	1.45 ± 0.28	<0.01	<0.01	NS
E/Ea avg	8.28 ± 2.96	8.41 ± 3.23	5.26 ± 0.49	<0.01	<0.01	NS
E/VP	1.65 ± 0.55	1.63 ± 0.61	1.34 ± 0.14	<0.05	<0.05	NS
PCWP (mmHg)	13.73 ± 4.35	13.92 ± 4.75	9.29 ± 0.72	<0.01	<0.01	NS
Sa avg (cm/s)	8.73 ± 1.69	10.10 ± 1.37	9.96 ± 1.82	<0.01	NS	<0.01

**Notes.**

P1 value between groups 1&3 P2 value between groups 2&3 P3 value between groups 1&2 LAV Left atrial volume AO aortic diameter IVST interventricular septum thickness PWT posterior wall thickness LVIDd left ventricular internal diameter at end-diastole LVIDs left ventricular internal diameter at end-systole LVM left ventricular mass FS Fraction shortening EF ejection fraction LVOTD Left ventricular outflow tract diameter LVOT VTI Left ventricular outflow tract velocity time integral SV Stroke volume COP Cardiac output MAPSE Mitral annular plane systolic excursion E Peak velocity of early filling A Peak velocity of atrial filling DT deceleration time IVRT Isovolumic relaxation time. LV Vp Left ventricular velocity of propagation AEF Atrial ejection force Ea avg average peak early diastolic annular velocity Aa avg average peak late diastolic annular velocity PCWP Pulmonary capillary wedge pressure Sa avg average peak systolic annular velocity

## Results

The demographic data of the patient groups (1 & 2) and the control group (3) revealed mean ages (54.90 ± 11.13), (50 ± 13.73) (51.55 ± 8.71) years, respectively. In group I, 19 were males (45.24%) and 23 were females, in group II, 22 were males (52.38%) and 20 were females (47.62%). In the control group, 10 were males (43.48%) and 13 (56.52%) were females (Table 1).

There was no significant difference between the patient groups and control regarding age & gender.

Laboratory data of the studied groups are shown in Table 2.

The echocardiographic findings (Table 3), show a significant increase in LAV (*P* < 0.01, *P* < 0.01), LVM (*P* < 0.01) in both patient groups compared to the control group. SV was significantly increased in group II compared to group I and III (*P* < 0.01) and in group I compared to group III (*P* < 0.05). COP was significantly higher in both patients’ groups compared to the control group and in group II compared to group I (*P* < 0.01). MAPSE was significantly decreased in both patients’ groups compared to the control group (*P* < 0.01, *P* < 0.05) and in group I compared to group II (*P* < 0.05) with a significant decrease in S velocity in group I compared to group II and III (*P* < 0.01). There was a significant decrease in Vp (*P* < 0.01) with a significant increase in AEF (*P* < 0.01) in both patients’ groups compared to the control group and AEF was significantly increased in group II compared to group I (*P* < 0.01). Ea velocity and Ea/Aa were decreased significantly (*P* < 0.01) with significant increase in Aa velocity (*P* < 0.05, *P* < 0.01), E/Ea (*P* < 0.01) and E/Vp (*P* < 0.05) in both patients’ groups compared to the control group.

There was a significant increase in hs-cTnT levels in both patient groups compared to control group (*P* < 0.01, *P* < 0.01) (Table 4).

**Table 4:** Comparison between hs-cTnT level of the studied groups.

Variables	**Group I** (*N* = 42) Mean ± SD	**Group II** (*N* = 42) Mean ± SD	**Group III** (*N* = 23) Mean ± SD	*P* value		
				1&3	2&3	1&2
hs-cTnT (ng/L)	36.83 ± 24.14	30.46 ± 19.33	3.90 ± 1.81	0.0001	0.0001	NS

**Notes.**

hs-cTnT High sensitivity cardiac troponin T

We found a positive correlation between hs-cTnT level and LAV ( *r* = 0.291, *p* < 0.03), IVST (*r* = 0.374, *p* < 0.004), PWT ( *r* = 0.309, *p* < 0.02), LVM (*r* = 0.282, *p* < 0.03), A-wave velocity (*r* = 0.271, *p* < 0.04), E/Ea (*r* = 0.506, *p* < 0.0001), PCWP (*r* = .507, *p* < 0.0001) and a negative correlation between hs-cTnT and MAPSE (*r* =  − 0.300, *p* < 0.02), S-wave velocity (*r* =  − 0.259, *p* < 0.05), Ea ( *r* =  − 626, *p* < 0.0001), Ea/Aa (*r* =  − 0.543, *p* < 0.0001) (Table 5).

**Table 5:** Correlation between hs-cTnT level and echocardiographic parameters.

	LAV	IVST	PWT	LVM	MAPSE	A velocity	Ea avg	Ea/Aa avg	E/Ea avg	PCWP	Sa avg
r	0.291	0.374	0.309	0.282	−0.300	0.271	−0.626	−0.543	0.506	0.507	−0.251
P	0.027	0.004	0.018	0.032	0.022	0.039	0.0001	0.0001	0.0001	0.0001	0.05

**Notes.**

LAV Left atrial volume IVST interventricular septum thickness PWT posterior wall thickness LVM left ventricular mass MAPSE Mitral annular plane systolic excursion A Peak velocity of atrial filling Ea avg average peak early diastolic annular velocity PCWP Pulmonary capillary wedge pressure Sa avg average peak systolic annular velocity

We found a positive correlation between hs-cTnT level and serum urea and creatinine in addition to a negative correlation between hs-cTnT and glomerular filtration rate (GFR) (Table 6).

**Table 6:** Correlation between hs-cTnT levels and urea, creatinine and GFR.

		Urea	Creatinine	GFR
hs-cTnT (ng/L)	r	0.494	0.423	−0.604
	P	0.0001	0.001	0.0001

**Notes.**

GFR Glomerular filtration rate

Receiver Operating Curves (ROC) were established to show the diagnostic performances of LA volume, IVST, LVPWT, LV mass, MAPSE, A, Ea average, Ea/Aa average, E/Ea average, PCWP, and S average m in comparison with Troponin regarding the studied groups.

### Discrimination of group I vs control group III

**Figure 1.**
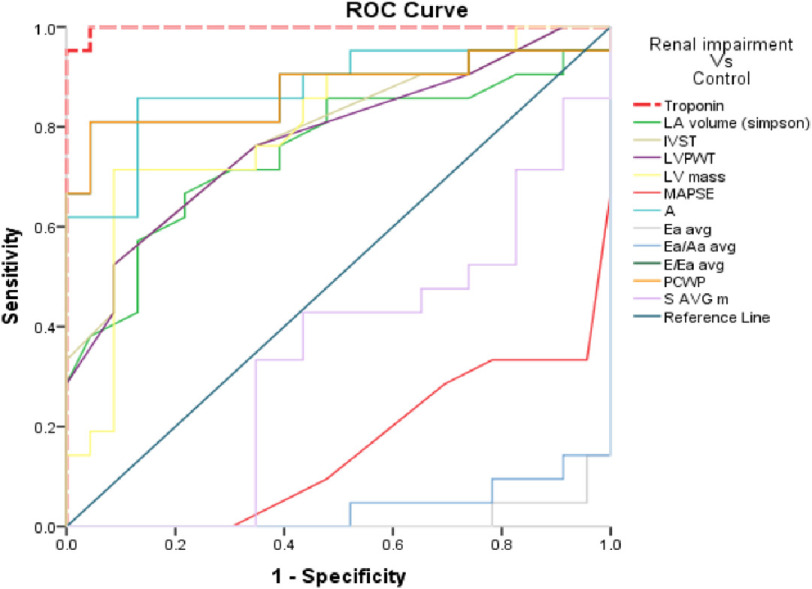
ROC Curve of the studied markers in regarding Group I Vs control group.

In reference to Figure 1 and Table 7 it was found that:

**Table 7:** Diagnostic performances of the studied markers among group I Vs control group.

**Studied groups**	**Studied markers**	**Cut-off**	**Sn.**	**Sp.**	**Accuracy**	**AUC**	**95% C.I**	***p*** **value**
**Group I** **Vs** **Control group**	Troponin	>5	100.0	95.0	95.65	0.998	0.991 - 1.000	<0.01
	LA volume	>44	66.67	78.26	44.93	0.754	0.601 - 0.871	<0.01
	IVST	>1.1	52.38	91.30	43.69	0.786	0.636 - 0.895	<0.01
	LVPWT	>1.1	52.38	91.30	43.69	0.777	0.627 - 0.889	<0.01
	LV mass	>213.88	71.43	91.30	62.73	0.798	0.650 - 0.904	<0.01
	MAPSE	≤13	66.67	95.65	62.32	0.844	0.703 - 0.935	<0.01
	A	>57.46	85.71	86.96	72.67	0.876	0.741 - 0.956	<0.01
	Ea avg	≤11.45	95.24	95.65	90.89	0.986	0.893 - 1.000	<0.01
	Ea/Aa avg	≤0.97	85.71	100.00	85.71	0.963	0.857 - 0.997	<0.01
	E/Eaavg	>5.76	80.95	95.65	76.60	0.874	0.739 - 0.955	<0.01
	PCWP	>10.01	80.95	95.65	76.60	0.874	0.739 - 0.955	<0.01

**Notes.**

Sn Sensitivity Sp Specificity AUC Area under curve C.I 95% Confidence Interval

*p* value <0.05 is significant, *p* value <0.01 is highly significant.

• Troponin at the cut-off value of >5 ng/L, revealed 100% sensitivity and 95% specificity with areas under curve (AUC) 0.998 and accuracy 95.65% (*P* < 0.01).
• LA volume at the cut-off value of >44 mL, revealed 66.67% sensitivity and 78.26% specificity with AUC 0.754 and accuracy 44.93% (*P* < 0.01).
• IVST and LVPWT at the cut-off values of >1.1, revealed 52.38% sensitivity and 91.30% specificity with AUC 0.786 & 0.777 respectively and accuracy 43.69% (*P* < 0.01).
• LV mass at the cut-off value of >213.88 gm revealed 71.34% sensitivity and 91.30% specificity with AUC 0.798 and accuracy 62.73% (*P* < 0.01).
• MAPSE at the cut-off value of ≤13 mm revealed 66.67% sensitivity and 95.65% specificity with AUC 0.844 and accuracy 62.32% (*P* < 0.01).
• A velocity at the cut-off value of >57.46 cm/s revealed 85.71% sensitivity and 86.96% specificity of with AUC 0.876 and accuracy 72.67%. (*P* < 0.01).
• Ea (average) at the cut-off value of ≤11.45 cm/s revealed 95.24% sensitivity and 95.65% specificity with AUC 0.986 and accuracy 90.89% (*P* < 0.01).
• Ea/Aa (average) at the cut-off value of ≤0.97 revealed sensitivity of 85.71% and specificity of 100% with AUC 0.963 and accuracy 85.71% (*P* < 0.01).
• E/Ea (average) at cut-off value of >5.76 revealed 80.95% sensitivity and 95.65% specificity with AUC 0.874 and accuracy 76.60% (*p* > 0.01).
• PCWP at cut-off value of >10.01 revealed 80.95% sensitivity and 95.65% specificity with AUC 0.874 and accuracy 76.60% (*P* < 0.01).

### Discrimination of group II Vs control group III

**Figure 2.**
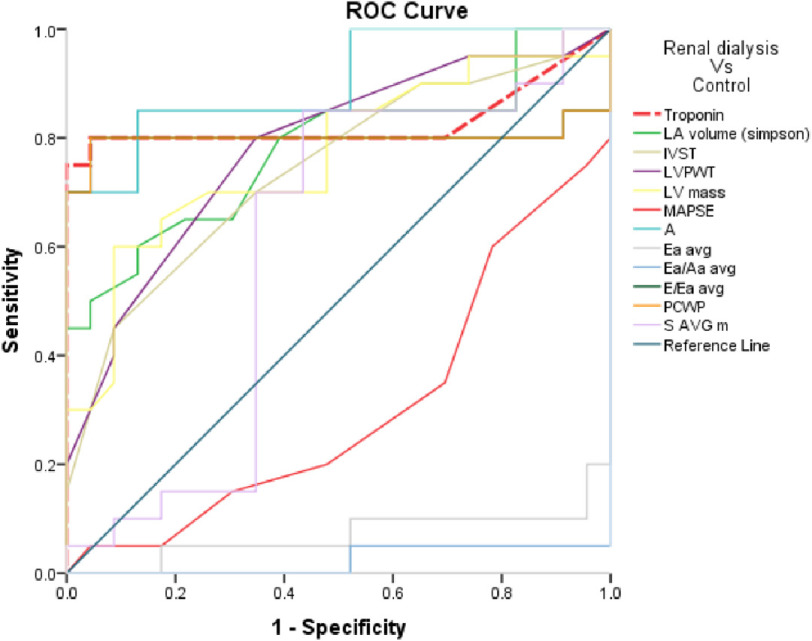
ROC Curve of the studied parameters in regarding Group II Vs control group.

With reference to Figure 2 and Table 8 we found that:

**Table 8:** Diagnostic performances of the studied markers among group II Vs control group.

**Studied groups**	**Studied markers**	**Cut-off**	**Sn.**	**Sp.**	**Accuracy**	**AUC**	**95% C.I**	***p*** **value**
**Group II** **Vs** **Control group**	Troponin	>5	76.2	95.7	71.84	0.796	0.638 - 0.954	<0.01
	LA volume	>46	61.90	86.96	48.86	0.789	0.639 - 0.897	<0.01
	IVST	>1.1	47.62	91.30	38.92	0.758	0.605 - 0.874	<0.01
	LVPWT	>1	80.95	65.22	46.17	0.792	0.643 - 0.899	<0.01
	LV mass	>213.88	61.90	91.30	53.21	0.777	0.627 - 0.889	<0.01
	MAPSE	≤15	61.90	69.57	31.47	0.682	0.525 - 0.814	<0.05
	A	>57.46	85.71	86.96	72.67	0.907	0.780 - 0.973	<0.01
	Eaavg	≤11.15	90.48	95.65	86.13	0.934	0.816 - 0.987	<0.01
	Ea/Aa avg	≤1.1	95.24	100.00	95.24	0.977	0.880 - 0.999	<0.01
	E/Eaavg	>5.76	80.95	95.65	76.60	0.810	0.663 - 0.912	<0.01
	PCWP	>10.01	80.95	95.65	76.60	0.810	0.663 - 0.912	<0.01

**Notes.**

Sn Sensitivity Sp Specificity AUC Area under curve C.I 95% Confidence Interval

*p* value <0.05 is significant, *p* value <0.01 is highly significant.

• It was found that, Troponin at the cut-off value of >5 ng/L, revealed 76.2% sensitivity and 95.7% specificity with AUC 0.796 and accuracy 71.84% (*P* < 0.01).
• LA volume at the cut-off value of >46 mL, revealed 61.90% sensitivity and 86.96% specificity with AUC 0.789 and accuracy 48.86% (*P* < 0.01).
• IVST at the cut-off value of >1.1, revealed 47.62% sensitivity and 91.30% specificity with AUC 0.758 and accuracy 38.92% (*P* < 0.01).
• LVPWT at the cut-off value of >1 revealed 80.95% sensitivity and 65.22% specificity with AUC 0.792 and accuracy 46.17% (*P* < 0.01).
• LV mass at the cut-off value of >213.88 gm revealed 61.90% sensitivity and 91.30% specificity with AUC 0.777 and accuracy 53.21% (*P* < 0.01).
• MAPSE at the cut-off value of ≤15 mm revealed 61.90% sensitivity and 69.57% specificity with AUC 0.682 and accuracy 31.47% (*P* < 0.05).
• A velocity at the cut-off value of >57.46 cm/s revealed 85.71% sensitivity and 86.96% specificity of with AUC 0.907 and accuracy 72.67%. (*P* < 0.01).
• Ea (average) at the cut-off value of ≤11.15 cm/s revealed 90.48% sensitivity and 95.65% specificity with AUC 0.934 and accuracy 86.13% (*P* < 0.001).
• Ea/Aa (average) at the cut-off value of ≤1.1 revealed sensitivity of 95.24% and 100% specificity with AUC 0.977 and accuracy 95.24% (*P* < 0.01).
• E/Ea (average) at cut-off value of >5.76 revealed 80.95% sensitivity and 95.65% specificity with AUC 0.810 and accuracy 76.60% (*P* < 0.01).
• PCWP at cut-off value of >10.01 revealed 80.95% sensitivity and 95.65% specificity with AUC 0.810 and accuracy 76.60% (*P* < 0.01).

### For discrimination of group I vs group II (Figure 3) (Table 9):

**Figure 3.**
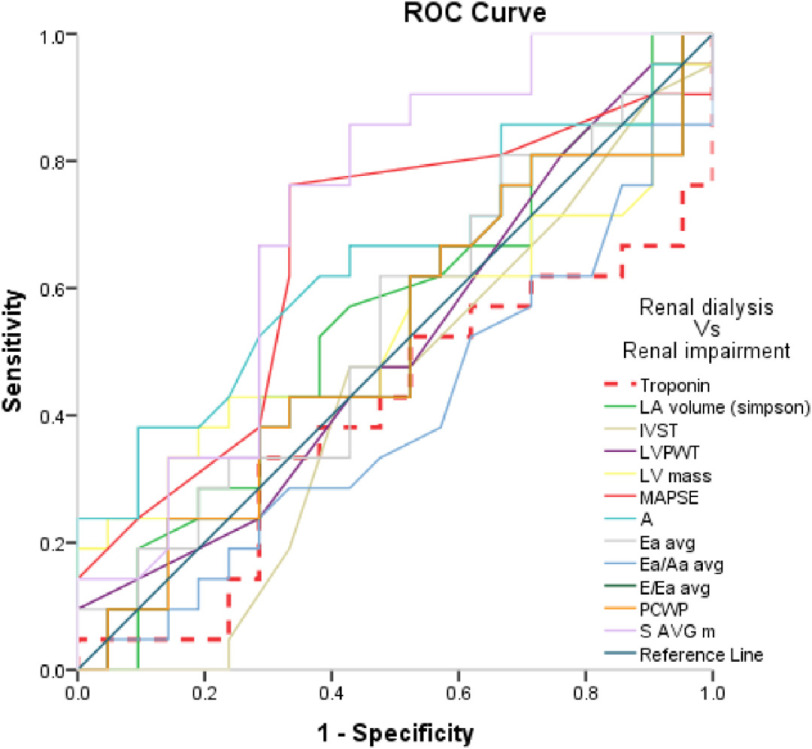
ROC Curve of the studied parameters in regarding Group I Vs Group II.

With refernce to Figure 3 and Table 9 we found that:

• Only average S-wave velocity and MAPSE revealed significance.
• Average S-wave velocity at the cut-off value of >9.11 cm/s revealed 86.96% sensitivity and 57.14 specificity with AUC 0.719 and accuracy 44.10% (*P* < 0.01).
• MAPSE at the cut-off value of >13 mm revealed 78.26% sensitivity and 66.67 specificity with AUC 0.668 and accuracy 44.93% (*P* < 0.05).

**Figure 4.**
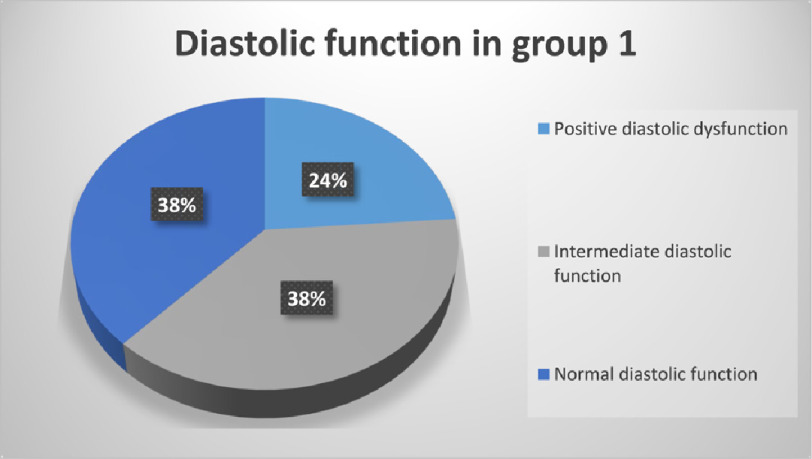
Diastolic function in group I.

Regarding diastolic function in group I (Figure 4), diastolic dysfunction defined by the current ASE/EACVI guidelines^15^ was present in 23.81%, normal diastolic function in 38.10% and it was intermediate or inconclusive in 38.10%.

**Table 9:** Diagnostic performances of the studied markers among group I vs group II.

**Studied groups**	**Studied markers**	**Cut-off**	**Sn.**	**Sp.**	**Accuracy**	**AUC**	**95% C.I**	***p*** **value**
**Group I** **Vs** **Control group**	MAPSE	>13	78.26	66.67	44.93	0.668	0.510 - 0.802	<0.05
	S avg	>9.11	86.96	57.14	44.10	0.719	0.564 - 0.844	<0.01

**Notes.**

Sn Sensitivity Sp Specificity AUC Area under curve C.I 95% Confidence Interval

*p* value <0.05 is significant, *p* value <0.01 is highly significant.

**Figure 5.**
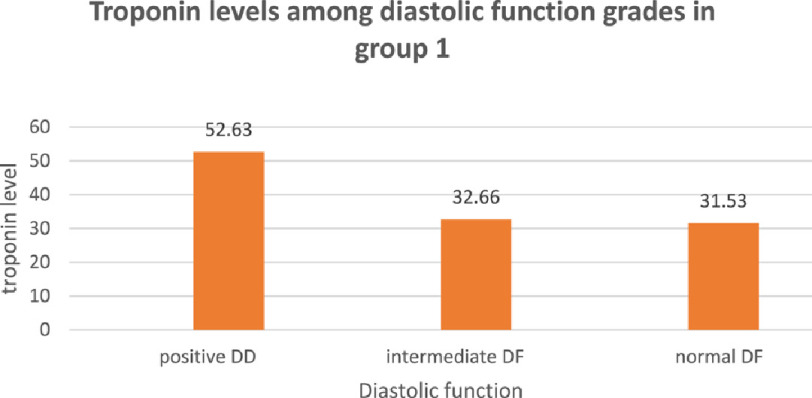
Troponin levels among diastolic function grades in group 1. DD, diastolic dysfunction; DF, diastolic function.

The mean troponin level was 52.63 ± 23.36 ng/L in patients with positive diastolic function, 32.66 ± 22.52 ng/L in patients with intermediate or inconclusive diastolic function and 31.53 ± 14.93 ng/L in patients with normal diastolic function (Figure 5). The difference in troponin levels was statistically significant in comparison between patients with positive diastolic dysfunction and patients with intermediate or normal diastolic function (*P* < 0.01) but the difference in troponin level between patients with intermediate and patients with normal diastolic function is statistically insignificant.

**Figure 6.**
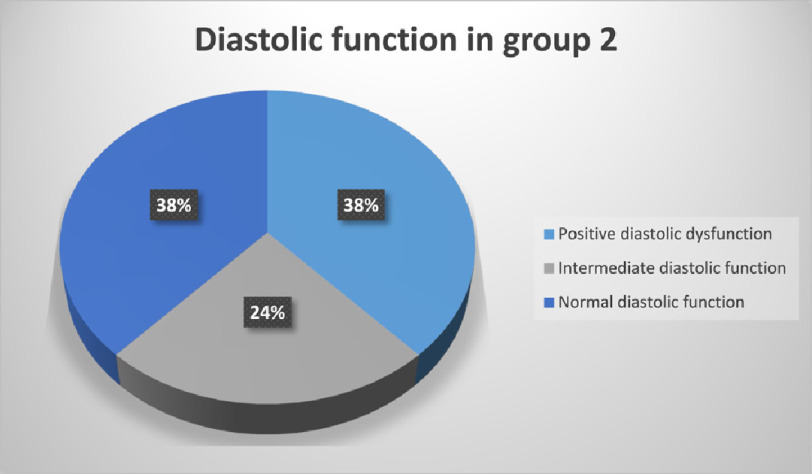
Diastolic function in group 2.

**Figure 7.**
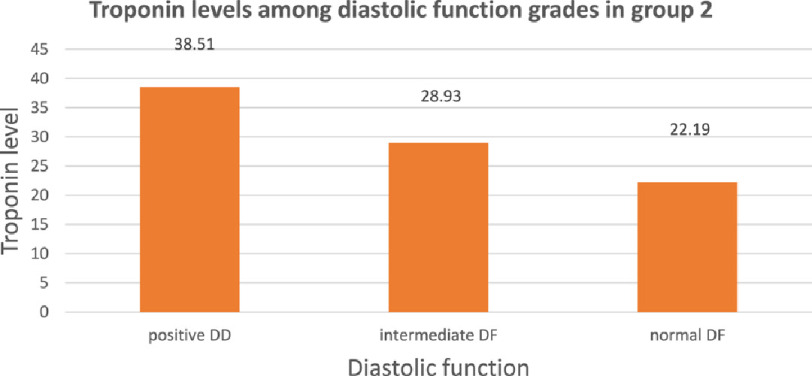
Troponin levels among diastolic function grades in group 1. DD, diastolic dysfunction; DF, diastolic function.

In group II, diastolic dysfunction was present in 38.10%, normal in 38.10% and inconclusive or intermediate in 23.80% of patients (Figure 6). The mean troponin level was 38.51 ± 31.35 ng/L in patients with positive diastolic function, 28.93 ± 20.34 ng/L in patients with intermediate or inconclusive diastolic function and 22.19 ±  15.50

ng/L in patients with normal diastolic function. The difference in troponin levels was statistically insignificant in comparison between patients with positive diastolic dysfunction and patients with intermediate diastolic function (*P* = 0.25). Also, the difference in troponin level between patients with intermediate and patients with normal diastolic function is statistically insignificant (*P* = 0.23). The difference in troponin levels was statistically significant in comparison between patients with positive diastolic dysfunction and patients with normal diastolic function (*P* < 0.05) (Figure 7).

## Discussion

This study investigated the association of circulating hs-cTnT with LV structural and functional abnormalities as detected by echocardiography among dialysis dependent and non-dialysis dependent CKD patients.

We demonstrate significant increase in left ventricular mass in both patients’ groups compared to the control group. This is in agreement with previous reports that estimated the prevalence of left ventricular hypertrophy (LVH) in CKD patients to range from 40% to 78%, to reach 75% at the time of initiation of dialysis^23,24^. de Simone reported that concentric LV hypertrophy is a natural pattern in end-stage renal disease (ESRD), and it identifies more severe impairment of the cardiovascular system^25^.

We found a significant increase in aortic root diameter (ARD) in both patient groups compared to the control group. Our results are in agreement with the results of Mulé et al., who found significantly increased ARD in hypertensive subjects with CKD when compared to those with normal renal function, in addition to significant and inverse association of GFR with ARD as assessed by transthoracic echocardiography (TTE)^26^.

In addition to the remodelling of the aortic root as a result of increased stress on the aortic wall due to hypertension, the cause of aortic root dilatation in CKD seems to be due to mechanisms similar to those responsible for decreased large artery elasticity in CKD patients, such as endothelial dysfunction, renin-angiotensin-aldosterone system and endothelin system activation, inflammation, oxidative stress and lipid peroxidation^27^. Aortic root dilatation may play a role in the increased cardiovascular risk associated with renal insufficiency. Cuspidi et al., concluded that LVH and ARD dilatation is a stronger predictor of cardiovascular events than LVH alone in general population^28^.

Our study revealed a significant increase of left ventricular internal diameter at end diastole (LVIDd) in the dialysis-dependent group II compared to the control. The increased LVIDd can be due to anemia, high-flow arteriovenous shunts, sodium, or water retention^29^.

Stroke volume (SV) was significantly increased in the dialysis-dependent group compared to other groups. COP was higher in both patient groups compared to the control group. It was also higher in the dialysis-dependent group compared to the non-dialysis dependent CKD patients.

The increased SV and COP in CKD patients is a consequence of anemia that leads to hemodynamic changes that act to raise COP including: decreased systemic vascular resistance, increased stroke volume, decreased blood viscosity, increased venous return and activation of the sympathetic nervous system. Also, the creation of arteriovenous shunts for hemodialysis access is partly responsible for high output states in dialysis dependent patients^30^.

Regarding systolic function, although we found no statistically significant differences in ejection fraction (FF%) and fraction shortening (FS) between groups, mitral annular plane systolic excursion (MAPSE) was significantly decreased in both patient groups compared to the control group. Additionally, we found a significant decrease in the peak systolic wave velocity (Sa) of the mitral annulus in the non-dialysis dependent CKD patients compared to the dialysis dependent group and the control group.

Although LVEF has been widely used to define systolic function, it gives limited insight into direction-based myocardial systolic function. MAPSE and S-wave velocity assess LV longitudinal function. Reduced LV longitudinal function is indicative of subclinical impairment of the systolic function^31^. Kai Hu et al., concluded that MAPSE is a sensitive echocardiographic parameter to assess global longitudinal LV function and seems to be more sensitive than EF for detecting early systolic dysfunction^32^.

Subclinical systolic dysfunction can be observed in patients with CKD despite normal LVEF. Impairment of LV longitudinal function reflects early CKD-related myocardial changes such as myocardial ischemia and interstitial fibrosis because sub-endocardial longitudinal myocardial fibers are more vulnerable to ischemia and increased wall stress^33,34^.

The results of the studies of Israa et al.,^35^ and Luszczak et al.,^36^ highlight the sensitivity of MAPSE to early changes in LV systolic function and concluded that MAPSE can be used as a sensitive tool to detect early longitudinal LV systolic dysfunction and in the absence of global longitudinal strain (GLS) by speckle-tracking echocardiography (STE).

The use of MAPSE by M- mode echocardiography and/or peak systolic velocity (Sa) of the mitral annulus by pulsed-wave Doppler tissue imaging (DTI) can be a reliable alternative for quantification of LV longitudinal function^35^.

Lui et al.,^37^ showed that GLS deteriorated along with the decline of renal function among CKD patients. In agreement with our study, Ravera et al.,^38^ reported that renal disease is associated with early and subclinical impairment of LV systolic function in spite of normal standard EF, regardless of the degree of renal function. Our finding of more impairment of LV longitudinal function in the non-dialysis dependent CKD patients compared to the dialysis dependent patients is consistent with the study of Lui et al., who reported better GLS in ESRD patients on dialysis than in those with CKD not on dialysis yet^37^.

Concerning left atrial (LA) function and diastolic function, our study showed a significant increase in left atrial volume (LAV) in both patients’ groups compared to the control group. The increased LAV may reflect both volume expansion and diastolic dysfunction in CKD patients. Additionally, there was a significant decrease in velocity of flow propagation (Vp) with a significant increase in atrial ejection force (AEF) in both patient groups compared to the control group. AEF is also significantly increased in the dialysis-dependent patients compared to the non-dialysis dependent CKD patients.

Vp is inversely related to the time constant of LV relaxation and is a preload independent index of LV diastolic performance^39,40^. AEF is the force exerted by the LA during atrial systole to push blood into the LV^41^ and it can be a useful index in assessing the atrial contribution to diastolic performance^42^. The rise in Left AEF in both patients’ groups may reflect the increase in the vigor of LA contraction with rising left ventricular end diastolic pressure (LVEDP).

Increased LA systolic force, was found to be associated with a significant increase in cardiovascular events^43^. Our results are in agreement with the results of Kadappu et al. ^44^ who demonstrated increased LAV and impaired LA strain in CKD patients. Similar findings have been reported by Tripepi et al. ^45^ who found increased LAV index in patients with ESRD. El-Sherbeny and El=hefnawy^46^ also reported LA dysfunction and enlargement in patients with early CKD and they found that the alteration in LA function (systolic strain) precede the changes in LV function (EF).

We found a significant increase in A-wave velocity with a significant decrease in E/A ratio in patient groups compared to the control group. DTI at the mitral annulus revealed significantly decreased Ea velocity and Ea/Aa ratio with a significant increase in Aa velocity, E/Ea, E/Vp and mean pulmonary capillary wedge pressure (PCWP) in both patient groups compared to the control group.

Our results show that left ventricular diastolic dysfunction is present in dialysis-dependent and non-dialysis dependent CKD patients. In agreement with our study, the study of Farshid et al. who concluded that some degree of diastolic dysfunction was present in 86% of patients on hemodialysis^47^. Also, the study of Matuso et al. on Japanese patients with ESRD showed that almost all patients had some degree of abnormal LV filling pattern^48^. A previous study demonstrated that the presence and severity of CKD is associated with the progression of LV diastolic dysfunction independently of age, sex, hypertension, coronary disease, and ejection fraction^49^.

The study of Sidmal et al. on patients with early and ESRD with or without dialysis showed that left ventricular diastolic dysfunction is present in all patients with CKD, including those with an early stage of CKD and they found that diastolic dysfunction got worsened in parallel with the severity of kidney dysfunction. They concluded that Doppler indices can detect subtle changes of diastolic function caused by CKD^50^.

Regarding hs-cTnT levels, our study revealed significant increase in hs-cTnT levels in both patient groups compared to the control group. In agreement with our findings, deFilippi et al., reported that patients with CKD have persistently elevated hs-cTnT levels compared with those with normal renal function^51^. We found a positive correlation between hs-cTnT level and LAV, IVST, PWT, LVM, A wave velocity, E/Ea ratio, PCWP and a negative correlation between hs-cTnT and MAPSE, S wave velocity, Ea, Ea/Aa ratio. Similar findings have been reported by Sun et al.,^52^ who found higher levels of hs-cTnT in non-dialysis CKD patients than in normal population and that the progressively higher hs-cTnT quartiles were associated with greater LVM index and higher prevalence of LV diastolic dysfunction.

In contrast to our study, Sun et al., found that hs-cTnT is associated with LVEF. We found no correlation between hs-cTnT level and EF but we found a negative correlation between hs-cTnT and MAPSE & Sa wave velocity of the mitral annulus, which measures LV longitudinal function.

In agreement with our findings, Liu et al. ^53^ found that asymptomatic ESRD patients with normal EF who have high hs-cTnT levels had more severe cardiac systolic dysfunction as determined by GLS than those with low hs-cTnT level. Other previous studies revealed that hemodialysis patients with increased hs-cTnT levels have more impaired LV systolic function, higher LV filling pressure (estimated from E:Ea and E:Vp) and higher LV mass index^54,55^.

Kang et al., in the Korean Cohort Study for Outcome in Patients with Chronic Kidney Disease^56^ reported that in CKD patients, hs-cTnT is strongly associated with left ventricular hypertrophy and diastolic dysfunction (E/Ea > 15 at the medial annulus) for both estimated glomerular filtration rate categories (≥60 or <60 mL/min per 1.73 m2) but was not associated with systolic dysfunction (EF < 50%).

The study of Stein et al., on mild to moderate CKD patients in the Chronic Renal Insufficiency Cohort (CRIC), revealed that hs-cTnT was strongly associated with measures of left ventricular structure and function as well as left atrial structure and concluded that hs-cTnT is associated with echocardiographic measurements of subclinical cardiovascular disease^57^. Kitagawa et al., found that hs-cTnT and E/Ea were significantly increased and that Ea was significantly decreased with increasing CKD stage in non-diabetic CKD patients. They suggested that hs- cTnT may be a useful biomarker of left ventricular diastolic dysfunction in non-diabetic CKD patients^58^.

In patients with CKD, detecting cardiac disease at an early stage would facilitate aggressive pharmacological and non-pharmacological treatment to reduce cardiovascular complications. Earlier intervention may be the best way to reduce the burden of CKD on the cardiovascular system^5^. Studies have demonstrated that adequate control of cardiovascular risk factors associated with CKD (such as diabetes, hypertension, proteinuria, dyslipidemia, smoking and obesity) appears to benefit the cardiovascular system. Lifestyle modifications to establish a healthier way of life are the first steps to slow the progression, and even enhance the regression, of CKD and reduce the risk of cardiovascular complications at the same time^59^.

Modulating cardiovascular risk factors specific to people with CKD (such as elevated homocysteine levels or oxidant stress, anemia, functional vitamin D deficiency, inflammation (CRP), hyperphosphatemia, sodium and water excess and electrolyte imbalance) could be important in decreasing cardiovascular complications^60^. Strategies to reduce CVD risk should be tailored to the individual and should be specific to their particular CKD stage^61^.

A limitation of our study is that patients with CKD had significantly higher systolic and diastolic blood pressures compared to the control arm, which may be one of the mechanisms by which CKD causes myocyte necrosis. This raises the possibility that our findings may be attributed to hypertension rather than CKD disease per se.

Other mechanisms by which CKD causes myocyte necrosis may include stable CAD, subendocardial ischemia, clinically silent micro-MI, direct myocardial toxicity from uremic toxins, hemodynamic overload, and hemodialysis-related stress. LVH, which is initially a compensatory adaptive response, can lead to cardio-myocyte damage with continual LV overload^62^.

Elevated cTn in CKD patients, likely due to kidney disease-related subclinical cardiac damage, may be exacerbated by reduced renal clearance of cTn^63^.

## Conclusion

Dialysis-dependent and non-dialysis dependent CKD patients have structural and functional (systolic & diastolic) cardiac abnormalities that can be assessed by echocardiography.

MAPSE and Sa wave velocity of the mitral annulus may be impaired despite normal EF and FS in CKD patients and may be used to detect early longitudinal LV systolic dysfunction.

Levels of hs-cTnT are increased in CKD patients and is associated with LVH, LAV and some of the echocardiographic parameters of LV systolic and diastolic dysfunction.

Our research suggests that serum hs-cTnT assay may be an important, simple, and possibly cost-effective test for the early screening of cardiac structure and function in CKD patients to provide evidence for early intervention.

## Conflicts of interest

The authors declare no conflicts of interest.

### Funding

The authors received no specific funding for this research.

## List of abbreviations

A: peak velocity of atrial filling
Aa: peak late diastolic mitral annular velocity
AEF: atrial ejection force
ARD: aortic root diameter
CKD: chronic kidney disease
COP: cardiac output
CVD: cardiovascular disease
DT: deceleration time
E: peak velocity of early filling
Ea: peak early diastolic mitral annular velocity
ESRD: end-stage renal disease
FF: ejection fraction
FS: fraction shortening
GFR: glomerular filtration rate
hscTnT: high-sensitivity cardiac troponin T
IVRT: isovolumic relaxation time
IVST: interventricular septal thickness
LAV: left atrial volume
LV: left ventricle
LVDD: left ventricular diastolic dysfunction
LVH: left ventricular hypertrophy
LVID: left ventricle internal dimension
LVIDd: left ventricular internal diameter at end-diastole
LVIDs: left ventricular internal diameter at end-systole
LVM: left ventricular mass
LVOT: left ventricular outflow tract
MAPSE: mitral annular plane systolic excursion
PCWP: pulmonary capillary wedge pressure
PWT: posterior wall thickness
Sa: mitral peak systolic annular velocity
SV: stroke volume
TDI: tissue Doppler imaging
Vp: flow propagation velocity
VTI: velocity time integral
